# Cell pyroptosis in health and inflammatory diseases

**DOI:** 10.1038/s41420-022-00998-3

**Published:** 2022-04-11

**Authors:** Yongqi Wu, Jing Zhang, Sihui Yu, Yan Li, Jinrong Zhu, Kai Zhang, Rongxin Zhang

**Affiliations:** 1 Guangdong Province Key Laboratory for Biotechnology Drug Candidates, Institute of Basic Medical Sciences and Department of Biotechnology, School of Life Sciences and Biopharmaceutics, Guangdong Pharmaceutical University, Guangzhou, 51006 China; 2 Department of Chemistry and Biology, Ryerson University, Toronto, ON M5B 2K3 Canada

**Keywords:** Cell death, Chronic inflammation

## Abstract

Inflammation is a defense mechanism that can protect the host against microbe invasion. A proper inflammatory response can maintain homeostasis, but continuous inflammation can cause many chronic inflammatory diseases. To properly treat inflammatory disorders, the molecular mechanisms underlying the development of inflammation need to be fully elucidated. Pyroptosis is an inflammation-related cell death program, that is different from other types of cell death. Pyroptosis plays crucial roles in host defense against infections through the release of proinflammatory cytokines and cell lysis. Accumulating evidence indicates that pyroptosis is associated with inflammatory diseases, such as arthritis, pneumonia, and colonitis. Furthermore, pyroptosis is also closely involved in cancers that develop as a result of inflammation, such as liver cancer, esophageal cancer, pancreatic cancer, and colon cancer. Here, we review the function and mechanism of pyroptosis in inflammatory disease development and provide a comprehensive description of the potential role of pyroptosis in inflammatory diseases.

## Introduction to programmed cell death and pyroptosis

Acute inflammation is the host’s natural defense against external and internal infection or injury. Failure to fight an infection or injury can lead to chronic inflammation and possibly the development of cancer. The presence of a link between inflammation and tumor growth was first suggested in the 19th century by the German physician Rudolf Virchow [1] due to the infiltration of leukocytes into the tumor microenvironment [2]. Inflammation in the tumor microenvironment facilitates the proliferation and survival of malignant cells and promotes angiogenesis and tumor metastasis. It also disrupts the response of the adaptive immune system to hormone treatments and chemotherapy.

Programmed cell death (PCD) plays critical roles in resisting external infection and maintaining internal homeostasis [3]. There are six main types of PCD, including apoptosis, necrosis, efferocytosis, pyroptosis, ferroptosis, and autophagy [4]. Pyroptosis, also known as inflammatory cell necrosis, is a novel type of PCD and inflammatory caspase-dependent cell death. It is distinct from other types of PCD. When pyroptosis occurs, pores form in the cell membrane, and cell lysis occurs, leading to the release of proinflammatory cytokines, such as interleukin-1β (IL-1β) and interleukin-18 (IL-18) [5, 6]. Pyroptosis can be stimulated by noninfectious stimuli and microbes such as Salmonella, Francisella, and Legionella [5] and plays essential regulatory roles in the elimination of bacteria from specialized neutrophils, macrophages, monocytes, and dendritic cells [7]. The phenomenon of pyroptosis was first described in 1992 [8] but was first termed in 2001 when researchers observed that bacteria-infected macrophages can cause rapid lytic cell death dependent on caspase-1 activity. The word “pyroptosis” comes from the Greek term, ‘pyro’, which means fire or fever, and ‘ptosis’, which means falling or dropping [9].

In recent years, pyroptosis has received increasing attention due to its association with inflammatory diseases. Many studies have shown that pyroptosis is not only involved in the development of atherosclerosis [10, 11], Alzheimer’s disease [12] and HIV-1 infection [13, 14] but also extensively involved in the occurrence and development of a variety of inflammatory diseases, including the progression of hepatitis to liver cancer, colonitis to colon cancer, and gastritis to stomach cancer. However, a thorough understanding of pyroptosis is still lacking. Therefore, it is important to study the role of pyroptosis in both health and disease. This will help us elucidate the pathogeneses of diseases and provide new therapeutic options for inflammatory diseases.

This review aims to link inflammatory diseases with pyroptosis, determine the influence of molecular mechanisms associated with the pyroptosis pathway on the occurrence and development of inflammatory diseases, and provide new ideas and effective targets for the prevention and treatment of inflammatory diseases.

### Insight into the pyroptosis pathways

**Fig. 1:**
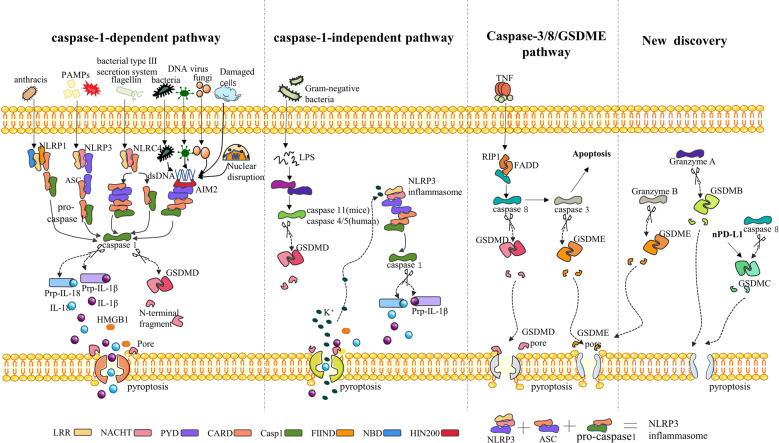
Molecular mechanism of pyroptosis. Early studies identified three pyroptosis pathways, including the caspase-1-dependent pathway, caspase-1-independent pathway and caspase-3/8/GSDMD pathway (left three panels). Recent studies have revealed a new pathway (right penal) in which granzyme A/B activate GSDMB/E to cause pyroptosis. In addition, nuclear PD-L1 can bind p-STAT3 to form a complex to activate and cleave GSDMC. Furthermore, Caspase-8 can cleave GSDMC, triggering pyroptosis. *PAMPs* pathogen-associated molecular patterns, *NLRP1/3* NLR pyrin domain-containing 1/3, *NLR* nucleotide-binding oligomerization domain, leucine-rich repeat-containing protein, *NLRC4* NLR CARD domain-containing 4, *ASC* apoptosis-associated speck-like protein containing a CARD, *CARD* caspase recruitment domain, *AIM2* absent in melanoma 2, *PYD* pyrin domain, *NBD* nucleotide-binding domain, *LRR* leucine-rich repeat.

According to recent studies, there are three pyroptosis pathways, i.e., the canonical inflammasome pathway (also called the caspase-1-dependent pathway), the noncanonical inflammasome pathway (also called the caspase-1-independent pathway) and the caspase-8/3/GSDME pathway [15] (Fig. 1).

In the canonical inflammasome pathway, NLRP1, NLRP3, NLRC4, AIM2, and other inflammasome sensors can detect foreign microbial signals and recruit the adaptor protein ASC to further recruit pro-caspase-1. Activated caspase-1 has two functions: 1) caspase-1 cleaves GSDMD and produces GSDMD-NT fragments, which can bind to phosphoinositides in the plasma membrane and then generate membrane pores and 2) caspase-1 is automatically cleaved to produce caspase-1 P10/P20 and P33/P10 tetramers, which catalyze the maturation of pro-IL-18 and pro-IL-1β into IL-18 and IL-1β, respectively, which are released into the extracellular matrix, causing inflammatory responses [16].

In the noncanonical inflammasome pathway, lipopolysaccharide (LPS) from gram-negative bacteria can activate caspase 11 in mice and caspase 4 and caspase 5 in humans. Subsequently, these activated caspases cleave GSDMD, and a pore is formed in the plasma membrane. The GSDMD pores allow potassium release, resulting in the activation of the NLRP3 inflammasome and IL-1β/IL-18 maturation. Moreover, GSDMD pores cause pyroptosis which can cause the release of mature cytokines [15].

In the Caspase-3/GSDME and Caspase-8/GSDMD pathways, TNF activates caspase-8 by interacting with other substances. Activated caspase-8 has two functions: it cleaves GSDMD to induce the formation of pores in the membrane and can activate caspase-3, which cleavages GSDME to induce the formation of pores in the membrane [15].

The latest research shows that in addition to the above three pathways, pyroptosis can be caused by a transition from apoptosis mediated by GSDME and that granzyme B can directly cleave GSDME at the same site as caspase-3 [17]. Granzyme A can also directly cleave GSDMB to cause pyroptosis [18]. PD-L1 can activate GSDMC through nuclear transfer to cleave gasdermin C and cause pyroptosis [19].

### The role of pyroptosis in health

Emerging evidence suggests that pyroptosis may act as an effective antimicrobial defense system in the host during infections [20]. First, pathogens can stimulate inflammasomes and cause pyroptosis, which can result in the lysis of infected cells and expose pathogens to extracellular defenses. This can expose pathogens to neutrophils to protect the host against infection [20]. Second, the inflammatory cytokines IL-1β and IL-18 can be secreted during the process of pyroptosis. IL-1β is not only a potent inducer of inflammation, vasodilation, and immune cell extravasation but also plays roles in shaping adaptive immune responses [21]. IL-18 promotes interferon (IFN)-γ production in Th1 cells, natural killer (NK) cells, and cytotoxic T cell, and promotes the development of Th2 cells and the local inflammatory response [22]. Such alarm signals also recruit immune cells to the site of infection for pathogen removal. Furthermore, pyroptosis can cause the release of extracellular inflammatory mediators, including IL-1 [5], heat-shock proteins [23] and ATP [24], which stimulate the production of proinflammatory cytokines via the activation of pattern-recognition receptors (PRRs). This “to die for life” signal helps the host control and clear microbial infections and allows tissues to return to a homeostatic state [5].

Inflammasomes are important in the process of pyroptosis. Dysfunction of inflammasomes may cause many diseases [25], such as Alzheimer’s disease and atherosclerosis [26]. The most common inflammasome is the NLRP3 inflammasome, which consists of NOD-like receptor family pyrin domain containing 3 (NLRP3), the inflammasome adaptor protein apoptosis-associated speck-like protein containing CARD (ASC), and pro-caspase-1. NLRP3 can be activated by a range of agonists, including ATP; pore-forming toxins; crystalline compounds; nucleic acids; hyaluronan; and fungal, bacterial, and viral pathogens. The formation of the NLRP3 inflammasome can cause pyroptosis and promote the maturation and secretion of IL-1β and IL-18 to protect organisms from invasive stress signals and pathogen infection and maintain health [27, 28]. A recent study reported that when pathogenic microorganisms are recognized by macrophages, these cells trigger an inflammatory response and release vimentin, which can promote the expression of Siglec-14. Siglec-14 can upregulate NLRP3 expression, promote the release of IL-1β, and cause pyroptosis [29].

Pyroptosis is related to the gasdermin (GSDM) family, which includes six members, namely, GSDMA, GSDMB, GSDMC, GSDMD, DFNA5 and DFNB59 [15]. GSDMD plays an especially important role in pyroptotic cell death. Thus, pyroptosis is also known as GSDM-mediated programmed necrotic death [16]. Therefore, GSDMs play a key role in maintaining health. Elucidating the molecular mechanism of pyroptosis is beneficial for the development of future strategies to prevent damage to the body by exogenous substances and maintain health.

Furthermore, apoptotic cell death is involved in resistance to several existing chemotherapeutic drugs. For example, decreased expression of GSDME enhances the resistance of melanoma cells to etoposide [30]. Therefore, the activation of nonapoptotic forms of programmed cell death may be an approach for the treatment apoptosis-resistant cancers. As a new form of cell death program, pyroptosis is promising as an effective target for treating apoptosis-resistant cancers. Currently, studies on the efficacy of targeting pyroptosis to treat inflammatory disease and cancer are limited.

However, some studies on pyroptosis have revealed that pyroptosis is both the cause of and the solution to the problem. On the one hand, moderate pyroptosis is helpful for eliminating infected cells in a timely manner, maintaining cell homeostasis, and effectively preventing excessive cell proliferation. In addition, inflammatory signals released as a result of pyroptosis, called “to die for life” signals, increase the immune response to further infection. On the other hand, a high level of pyroptosis may lead to aggravation of inflammatory symptoms and cause cell death and serious tissue and organ failure. These processes are related to the pathogenesis of some diseases and homeostasis disorders in vivo [11].

### Biological role of pyroptosis in inflammatory disease

Studies have shown that the chronic inflammatory response is not only related to tumor progression but also plays important roles in tumor immunity and immunotherapy [31]. Excessive pyroptosis can lead to a continuous inflammatory response, increased levels of the inflammatory mediators IL-1β, IL-18, and HMGB-1, and disruption of host homeostasis. It has been reported that GSDME-mediated pyroptosis leads to activation of the ERK1/2 pathway through the release of high-mobility group box protein 1 (HMGB1), promotes tumor cell proliferation and the expression of proliferating nuclear antigen (PCNA), and subsequently induces the development of colitis-associated colorectal cancer (CAC) [32]. In addition, pyroptosis is a caspase-dependent type of cell death that can be activated by inflammasomes. Moreover, caspase-1/4/5/11 are associated with pyroptosis and are involved in the pathogenesis of a variety of diseases, including hepatitis, inflammatory bowel disease, vascular inflammation [33], and myocardial infarction [34]. Studies have shown that some cancer cells develop chemotherapy resistance, anti-apoptotic ability and the ability to survival under stress conditions [35]. It is meaningful to study the therapeutic effects of a combination of drugs and pyroptosis-targeting strategies inflammatory diseases. The research progress on the association between pyroptosis and various inflammatory diseases is briefly discussed in Table 1.

**Table 1:** Pyroptosis is closely related to the progression of inflammation to cancer.

Molecule/drug	Cancer type	Biological function	Mechanism	Refs
SMS1	NASH	Suppression of tumor growth	Activates the NLRC4 inflammasome and caspase-1	[63]
GSDMD	NASH	Induction of cancer	Activates NF-kB and causes steatosis and cell pyroptosis	[62]
E2	HCC	Induction of cancer	Activates the NLRP3 inflammasome	[66]
HBx	Hepatitis B	Induction of cancer	Activates the NLRP3 inflammasome and promotes pyroptosis	[58]
HCV	Hepatitis C	Induction of canc	Promotes pyroptosis	[65]
Sorafenib	HCC	Suppression of tumor growth	Induces macrophage (MΦ) pyroptosis	[67]
BI2536	ESCC	Inhibition of cancer	Activates GSDME and induces pyroptosis	[86]
Metformin	ESCC	Suppression of tumor growth	Promotes pyroptosis	[88]
GSDMD	BE	Induction of cancer	Secretes inflammatory cytokines and chemokines	[82]
Alcohol	Esophagitis	Induction of cancer	Activates caspase-1	[81]
GSDMD	Gastric cancer	Suppression of tumor growth	Promotes pyroptosis	[83]
GSDME	Gastric cancer	Inhibition of tumor growth	Activates caspase-3 and cause pyroptosis	[87]
MST1	PDAC	Suppression of tumor growth	Induces pyroptosis via ROS	[84]
KJL	UC	Alleviation symptoms	Inhibits the occurrence of pyroptosis	[75]
HMGB1	CAC	Induction of cancer	Cleaves GSDME and releases HMGB1	[32]
IRGM	Colitis	Suppression of tumor growth	Inhibits NLRP3 inflammasome activation and pyroptosis	[76]
NALP1	Colon cancer	Suppression of tumor growth	Triggers pyroptosis	[79]
LXRb	Colon cancer	Inhibition of tumor growth	Induces pyroptosis by activating caspase-1	[77]
lobaplatin	Colon cancer	Inhibition of cancer	Regulates GSDME and trigger pyroptosis	[78]

### Pyroptosis in arthritis

Arthritis is a chronic inflammatory disease. Studies have shown that pyroptosis is closely related to arthritis (Table 2). In a study on gout, it was proven that BF-2 can inhibit NLRP3 inflammasome assembly and activation by blocking the binding of ASC and pro-caspase1. This reduces IL-1β secretion and inhibits pyroptosis in macrophages [36]. In study on osteoarthritis (OA), hydrogen peroxide (H_2_O_2_) treatment was found to increase the expression of ubiquitin-specific protease 7 (USP7). Experimental evidence has shown that USP7 expression is positively correlated with the level of NAD(P)H oxidase (NOX)4, which can increase reactive oxygen species (ROS) levels, stimulate NLRP3 activation and increase pyroptosis [37]. In addition, loganin inhibits NF-κB signaling and reduces pyroptosis in chondrocytes and may be used for OA treatment [38]. Additionally, licochalcone A (Lico A) inhibits the NF-κB pathway, activates Nrf2, and reduces the expression of NLRP3 inflammasome components, such as ASC and pro-caspase1. Therefore, Lico A can reduce chondrocyte pyroptosis and alleviate OA [39]. In a study on rheumatoid arthritis (RA), pentaxin 3 (PTX3) was found to promote RA monocyte pyroptosis in a C1q-dependent manner [40]. In contrast, intra-articular monosodium urate (MSU) crystals increase bromodomain-containing protein 4 (BRD4) expression, which can cause NLRP3 activation and promote macrophage pyroptosis. Furthermore, BRD4 activates the NF-κB pathway and promotes the inflammatory response. Consistently, the BRD4 inhibitor JQ-1 can be used to alleviate inflammation [41]. The presence of a large amount of acid leads to acidosis in vivo, which induces the activation of acid‐sensing ion channel 1a and causes articular chondrocyte pyroptosis through the calpain 2/calcineurin pathway, further leading to RA development and progression [42, 43].

**Table 2:** Cell pyroptosis in arthritis.

Disease	Stimulant	Molecular target	Function	Ref
Gout	BF-2	NLRP3 inflammasome	Activates NLRP3 and reduces pyroptosis	[36]
OA	H2O2		Promotes pyroptosis.	[37]
OA	Lico A		Inhibits the NF-κB pathway and pyroptosis	[39]
RA	BRD4		Actives NLRP3 and the NF-κB pathway	[41]
Arthritis	Gallic acid		Reduces the secretion of IL-1β	[48]
Arthritis	Wedelolactone		Inhibits pyroptosis	[49]
OA	Loganin	Caspase-1	Inhibits pyroptosis	[38]
Arthritis	P2Y14R		Increases pyroptosis	[44]
RA	ASIC1a		Causes pyroptosis	[42]
Arthritis	GSDMD SiRNA	GSDMD	Inhibits pyroptosis	[47]
sJIA	——	GSDMD-NT	Triggers pyroptosis	[50]

In the MSU-induced acute gouty arthritis model, the P2Y14 receptor (P2Y14R) activates the NLRP3 inflammasome, promotes caspase-1 expression, and increases pyroptosis in macrophages. In addition, NLRP3 interacts with cAMP to promote gout flare. The adenylate cyclase activator (forskolin) also causes pyroptosis [44, 45]. Based on these findings, P2Y14R antagonists are expected to inhibit the occurrence of pyroptosis and relieve acute gouty arthritis [46]. Decreasing GSDMD expression can alleviate MSU-induced acute gouty arthritis [47]. Gallic acid increases the expression of nuclear factor Nrf2 to inhibit the activity of the NLRP3 inflammasome, reduces the release of inflammatory factors, and alleviates gouty arthritis caused by MSU accumulation [48]. Wedelolactone can phosphorylate Ser/Thr residues of NLRP3 and inhibit the activity of the NLRP3 inflammasome, thereby inhibiting the occurrence of macrophage pyroptosis and alleviating MSU-induced arthritis [49].

### Pyroptosis in pneumonia

Since the global outbreak of COVID-19 at the end of 2019, numerous studies have shown that COVID-19-induced pneumonia is related to the massive accumulation of inflammatory cytokines in local organs. Therefore, an increasing number of studies are focusing on the associated between COVID-19 and pyroptosis because pyroptosis can cause inflammatory cytokine release [50–52]. Considering the important role of the NLRP3 inflammasome in the occurrence of pyroptosis, some researchers have proposed the use of nutrients that can inhibit inflammasome activity [53].

In a study on pneumonia, Maxing Shigan decoction (MXSG) was found to have a similar effect as the NLRP3 inhibitor INF39 and to downregulate NLRP3 expression and reduce IL-1β and IL-18 secretion [54]. In addition, activation of the IL-17 pathway can promote pyroptosis [55]. In a study on *Pseudomonas aeruginosa* (PA), which can induce acute lung injury, peptidylarginine deiminase (PAD)2 expression was found to be positively correlated with the expression of caspase-1. Inhibiting PAD2 expression was observed to effectively reduce macrophage pyroptosis and enhance bacterial clearance [56].

### Pyroptosis promotes the progression of chronic hepatitis to hepatocellular carcinoma

Liver disease is a serious health problem worldwide. Liver disease can develop into liver fibrosis (LF), cirrhosis and hepatocellular carcinoma (HCC). Recent studies have reported that pyroptosis is involved in the development and regulation of liver diseases [57].

**Fig. 2:**
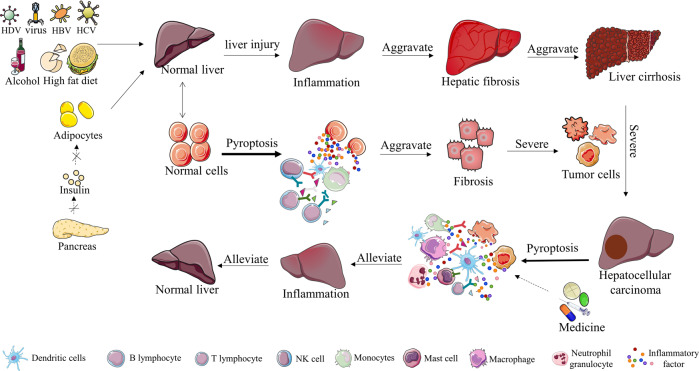
Chronic inflammation of the liver and its pathological development. Hepatitis progresses to liver cancer. Initially, virus invasion, alcohol accumulation, and lack of fat consumption can lead to liver damage and inflammation. Inflammation can also be caused by pyroptosis of liver cells. In this stage, immune surveillance can effectively prevent the deterioration of inflammation. However, if inflammation is not controlled, it can further damage liver function, leading to fibrosis, cirrhosis, and liver cancer. In hepatocellular carcinoma, stimulation of pyroptosis in liver cancer cells can enhance the immune response to a certain extent. In brief, the numbers of NK cells, macrophages, B lymphocytes, T cells, and other immune cells in the tumor microenvironment increase, and the levels of immune-stimulating molecules, such as IL-2, IL-12, IL-33, TNF-α, TNF-β, and IFN-γ, also increase. Combinations of chemotherapy drugs can kill more tumor cells and relieve disease symptoms.

Nonalcoholic fatty liver disease (NAFLD) can be further categorized into nonalcoholic fatty liver (NAFL) and nonalcoholic steatohepatitis (NASH) according to histology. NASH is a key step in the progression of NAFLD, which can further progress to fibrosis, cirrhosis, and hepatocellular carcinoma [58, 59] (Fig. 2). In the NAFL stage, the inflammation caused by pyroptosis is not obvious, and global damage is relatively mild. NLRP3 induces caspase-1 cleavage in macrophages and adipose tissue after sensing intracellular lipid toxicity-associated ceramide [60]. In contrast, in NASH, the inflammation caused by pyroptosis is much more serious and causes greater damage to the body. Bing Xu et al. confirmed that GSDMD plays a role in NASH. GSDMD activates NF-kB by regulating the secretion of IL-1β, subsequently causing steatosis, cell pyroptosis and an inflammatory response [61]. Interestingly, pyroptosis can promote NASH, but also relieves NASH. Eun Hee Koh et al. showed that sphingomyelin synthase 1 (SMS1) is highly expressed in NASH and that free cholesterol (FC) can induce the expression of SMS1. Diacylglycerol (DAG) produced by SMS1 can activate protein kinase Cδ (PKCδ), which further activates the NLR family CARD domain-containing protein 4 (NLRC4) inflammasome. The NLRC4 inflammasome activates Caspase-1 via the canonical inflammasome pathway of pyroptosis, triggering cell pyroptosis, thus forming a novel SMS1-DAGPKCδ-NLRC4 axis, which induces hepatoma pyroptosis [62].

Hepatitis can be divided into viral hepatitis and autoimmune hepatitis. There are two main types of viral hepatitis, hepatitis B (HB) and hepatitis C (HC). In an experimental study on hepatitis B, HBx was shown to be a leading mediator of hepatic inflammation [63]. Under oxidative stress, HBx activates the NLRP3 inflammasome in liver cells by inducing mitochondrial damage and producing mitochondrial reactive oxygen species (mitoROS) to trigger pyroptosis, ultimately causing the release of the proinflammatory mediator apoptosis-associated speck-like protein containing a caspase recruitment domain (ASC), IL-1β, IL-18, and high-mobility group box 1 (HMGB1) [57]. In a study on hepatitis C, H. M. Kofahi et al. showed that HCV-infected or bystander cells can induce pyroptosis mediated by the caspase-1 and caspase-3 signaling pathways, which is closely related to the pathogenesis of hepatitis C [64].

The most severe stage of liver disease is HCC; Thus, interventions for liver cancer are needed. Qing Wei et al. found that 17β-estradiol (E2) can induce the activation of the NLRP3 inflammasome and cause the occurrence of caspase-1-dependent pyroptosis in liver cancer cells, subsequently reducing autophagy, which is protective [65]. By combining a pyroptosis-target strategy with drugs, Carina Hage et al. showed that sorafenib, a broad-spectrum kinase inhibitor, can be used to treat hepatocellular carcinoma (HCC). Sorafenib induces macrophage pyroptosis, further releasing inflammatory signals that activate NK cells, enhance the effector function of NK cells and ultimately lead to tumor cell death [66]. Taxifolin (TAX) inhibits caspase-1 activity and IL-1β expression in steatotic liver cells, thereby inhibiting the occurrence of pyroptosis. Furthermore, TAX can regulate the expression levels of sterol regulatory element-binding protein-1 (SREBP1) and peroxisome proliferation–activated receptor gamma (PPARγ) to inhibit lipid accumulation and alleviate the inflammatory response [67]. Moreover, berberine (BBR) [68], baicalin (BA) [69], and liraglutide [70] can be used for the treatment of NASH. The commonality of these three substances is that they can inhibit the expression of NLRP3 and reduce the expression level of GSDMD, thus reducing pyroptosis and alleviating NASH [68–70].

### Pyroptosis promotes the development of chronic colitis to intestinal cancer

Long-term exposure to chronic inflammation can cause inflammatory bowel disease (IBD) such as ulcerative colitis (UC) and Crohn’s disease (CD). Colitis-associated colon cancer (CAC), a particularly aggressive subtype of colorectal cancer (CRC), occurs in patients with IBD and is considered closely associated with chronic inflammation, which is present in the early stages of tumor onset [71].

Carvalho et al. and Carvalho et al. found that IL-1β and IL-18 are the major inflammatory factors that induce UC and that pyroptosis is closely related to UC [72, 73]. Thus, inflammation can be alleviated by inhibiting the effect of pyroptosis. Kuijieling (KJL) reduces the expression levels of NLRP3, ASC, caspase-1, GSDMD-N, IL-1β, and IL-18 [74]. Subhash Mehto et al. used dextran sodium sulfate (DSS) to induce colitis in mice and verified that IRGM/Irgm1 can limit the activity of the NLRP3 inflammasome [75]. These findings show that regulatory molecules can inhibit pyroptosis and alleviate inflammatory symptoms during pyroptosis.

In CAC, activation of GSDME causes pyroptosis and the release of HMGB1 inducing tumor cell proliferation and proliferating cell nuclear antigen (PCNA) expression through the ERK1/2 pathway and further promoting the development of CAC [32]. Liver X receptor β (LXRβ) in the cytoplasm can interact with Pannexin-1 and specifically induce caspase-1 activation, which can induce pyroptosis in colon cancer cells [76]. Lobaplatin is a third-generation platinum antineoplastic agent with the strong antitumor effect that induces pyroptosis in colorectal cancer (CRC) cells by mediating GSDME in colon cancer cells [77]. Nucleotide-binding oligomerization domain-like receptor family pyrin domain-containing 1 (NALP1) is related to cell pyroptosis, and 5-aza-2-deoxycytidine (DAC) can restore the expression of NALP1 across and inhibit the growth of colon cancer cells [78].

### Pyroptosis is a key process in the progression of other inflammatory conditions to cancer

Gastrointestinal (GI) disorders occur when the balance among the microbiota in the GI tract is disrupted. In a study on esophageal adenocarcinoma (EAC), the composition of the intestinal flora of high-fat diet (HFD) induced BE model mice was altered, leading to inflammation and further progression to EAC [79]. In recent years, studies have shown that the inflammasome can protect the GI tract against invasive pathogens and maintain intestinal homeostasis. Pyroptosis occurs in esophagitis, amplifies inflammatory signals, and further accelerates esophagitis development. In addition, alcohol accumulation can activate caspase-1 and stimulate the maturation of the inflammatory cytokines IL-1β and IL-18, which causes pyroptosis in esophageal epithelial cells [80]. However, in addition to exerting these detrimental effects, pyroptosis plays roles in maintaining health by mediating the expression molecules in the pyroptosis pathway during disease. Caspase-1 mediates the inflammatory disease BE, which can further develop into EAC. Barber et al. showed that the secretion of inflammatory cytokines and chemokines is reduced in BE patient biopsies and in mouse BE organ cultures in vitro via inhibition of caspase-1 [81]. The expression of caspase-activated GSDMD is beneficial for inhibiting the proliferation of gastric cancer cells. GSDMD may regulate the cell cycle by suppressing the S to G2/M phase transition in gastric cancer cells through activation of the extracellular signal-regulated kinase (ERK), signal transducer and activator of transcription 3 (STAT3) and phosphatidylinositol 3 kinase/protein kinase B (PI3K/AKT) signaling pathways [82]^.^ In a study on pancreatic adenocarcinoma, mammalian STE20-like kinase 1 (MST1) was found to suppress the progression of pancreatic ductal adenocarcinoma (PDAC) cells through reactive oxygen species (ROS) induced pyroptosis [83]. A study showed that the levels of inflammatory markers are correlated with the prognosis of gastric cancer [84]. Therefore, we can identify inflammatory markers associated with pyroptosis and explore the relationship between them.

The chemotherapy drug cisplatin (DDP) combined with low-dose BI2536, a polo-like Kinase 1(PLK1) inhibitor, can increase chemosensitivity by inducing pyroptosis. This leads to a better curative effect and activation of Bax (an apoptosis-related protein) and Caspase-3, further inducing a switch from apoptosis to pyroptosis via GSDME [85]. Additionally, Yubin Wang et al. demonstrated that treating gastric cancer cells SGC-7901 or MKN-45 with a combination of 5-FU can increase GSDME expression and induce a switch from caspase-3-dependent apoptosis to pyroptosis in gastric cancer cells [86]. Similar studies have shown that metformin can downregulate proline-, glutamic acid- and leucine-rich protein-1 (PELP1) expression by increasing miR-497 expression, ultimately inducing ESCC cell pyroptosis. This suggests that metformin may be a therapeutic option for diseases that are resistant to chemotherapy and radiotherapy but sensitive to pyroptosis [87].

### Summary

Chronic inflammation is involved in the development of various kinds of diseases. Therefore, understanding the mechanism of inflammation progression is very important for preventing the occurrence and development of inflammatory diseases. Particularly, persistent chronic inflammation leads to the occurrence of cancer. For example, liver injury can cause hepatitis, liver fibrosis, and cirrhosis and ultimately lead to HCC [88]. Long-term damage to the esophageal mucosa results in gastroesophageal reflux disease, which further develops into Barrett’s esophagus or esophageal adenocarcinoma [89]. Prolonged colitis can lead to IBD and eventually to CRC.

Inflammasomes play essential regulatory roles in inflammation and pyroptosis [25] [28]. During pyroptosis, a series of pathogen-associated molecular patterns (PAMPs) stimulate inflammasomes, subsequently activating caspase-1/4/5/11 and inducing the production of GSDMD-N, which eventually leads to cell lysis, intracellular content release, and inflammatory cytokine production. Therefore, the release of IL-1β and IL-18 and increased GSDMD-N expression are involved in the occurrence and development of various inflammatory diseases. In addition, the NLRP3 inflammasome is one of the most important molecules in the process of pyroptosis. Accurate regulation of inflammasomes is a potential treatment strategy for inflammatory diseases. Inhibition of pyroptosis in normal cells can ameliorate inflammatory symptoms. However, pyroptosis in cancer cells can be regulated by drugs to kill these cells [68]. Therefore, it is meaningful to explore the molecular mechanism of pyroptosis in both healthy and tumor cells. Regulating key molecules involved in pyroptosis may help increase the death of tumor cells, decrease normal cell death, alleviate inflammation, and prevent the occurrence of inflammatory diseases.

Although pyroptosis has been demonstrated to be closely correlated with the progression of inflammatory diseases, many questions related to pyroptosis, such as whether NLRP3 inflammasome inhibitors are suitable for clinical application, are still unclear and need to be further explored; however, repairing membrane pores formed during pyroptosis is effective in the treatment of inflammatory diseases. Indeed, pyroptosis acts as a double-edged sword. On the one hand, inflammatory cytokines released during the process of pyroptosis can cause local inflammation, leading to the recruitment and activation of immune cells, which ultimately help the host clear the pathogen. This phenomenon also enhances immunity. In the treatment of cancer, the proliferation and migration of cancer cells can be inhibited by inducing cancer cell pyroptosis. Thus, developing activators or inhibitors of key molecules involved in pyroptosis and combining them with other immunotherapies to achieve a better therapeutic effect is a promising approach. On the other hand, during the development of inflammatory diseases, excessive activation of pyroptosis aggravates inflammatory responses and causes organ damage. NF-κB is known to be an upstream activator of the NLRP3-inflammasome and induces NLRP3 expression by triggering the priming and assembly of inflammasome [90]. Loganin inhibits chondrocyte pyroptosis and reduces aberrant angiogenesis via inhibition of NF-κB activation [38]. This suggests that activation of other pathways affects the occurrence of pyroptosis and then regulates the development of inflammatory diseases. Currently, immune checkpoint inhibitors are also used as immunotherapies [91]. As mentioned above, when hypoxia occurs, PD-L1 enters the nucleus and forms a complex with P-Y705-STAT3, resulting in apoptosis and pyroptosis [19]. Thus, should we combine drugs targeting immune checkpoint proteins (PD-L1) and pyroptosis for the treatment of cancer?

In conclusion, a comprehensively understanding of the physiological roles of pyroptosis and pyroptosis-related pathological mechanisms underlying the occurrence and development of inflammatory diseases is needed. Such an understanding will provide new strategies for the prevention and regulation of inflammatory diseases and new clinical approaches and ideas for the treatment of cancer.

## Supplementary information

Author Contribution Statement

## Data Availability

The data used to support the findings of this study are available from the corresponding author upon request.
