# Supplementary material for: Cell pyroptosis in health and inflammatory diseases
Source: Cell Death Discov. 2022 Apr 11;8:191. doi: 10.1038/s41420-022-00998-3 (PMC8995683; doi:10.1038/s41420-022-00998-3)
Supplement: Supplementary file 1 — Author Contribution Statement [file 41420_2022_998_MOESM1_ESM.doc]

Rongxin Zhang, Ph.D. Professor

Dept. of Guangdong Province Key Laboratory for Biotechnology Drug Candidates, Institute of Basic Medical Sciences and Department of Biotechnology, School of Life Sciences and Biopharmaceutics, Guangdong Pharmaceutical University, Guangzhou 510006, China

Tel: 0086-22-83336563 (O), 0086-22-83336561 (L)

E-mail: [rongxinz@yahoo.com](mailto:rongxinz@yahoo.com)

To: Editor, *Cell Death Discovery*

Re: Author contribution statement

March 21, 2022

Dear Editor,

This is the author's contribution statement of our manuscript. In this manuscript entitled “**Cell pyroptosis in health and inflammatory diseases**”, Y Wu drafted the manuscript. Y Wu, J Zhang, Y Li and S Yu discussed the manuscript. Y Wu and R Zhang designed the research and drafted the manuscript. J Zhu, K Zhang and R Zhang revised the manuscript. All authors read and approved final manuscript.

Sincerely yours,

Rongxin Zhang, Ph.D., Professor

Guangdong Pharmaceutical University
